# Stroke-like presentation of a small tuberculum sellae meningioma unmasked by corticosteroid use: A case report

**DOI:** 10.1016/j.radcr.2025.08.097

**Published:** 2025-09-29

**Authors:** Christoph Mader, Marcus Czabanka, Elke Hattingen, Christophe T. Arendt

**Affiliations:** a Institute of Neuroradiology, University hospital Frankfurt, Schleusenweg 2-16, 60528, Frankfurt am Main, Germany; b Department of Neurosurgery, University hospital Frankfurt, Schleusenweg 2-16, 60528, Frankfurt am Main, Germany

**Keywords:** Meningeoma, Stroke, Endocrine dysfunction, Corticosteroids

## Abstract

Tuberculum sellae meningiomas are known to cause visual symptoms due to their proximity to the optic apparatus. However, their impact on pituitary function, particularly when the tumor is small, is less frequently described. Although secondary adrenal insufficiency is rare in this context, it may be underrecognized. We report a case in which a small (1.2 cm) tuberculum sellae meningioma resulted in clinically relevant adrenocorticotropin deficiency, unmasked by intra-articular corticosteroid injections. The patient initially presented with nonspecific symptoms and transient ischemic attack-like episodes, which led to imaging and identification of the tumor. Surgical resection of the tumor resulted in complete resolution of symptoms. This case highlights the need for a high index of suspicion for pituitary dysfunction even in small suprasellar tumors and suggests that exogenous corticosteroids may exacerbate underlying hypothalamic-pituitary-adrenal axis compromise.

## Introduction

Suprasellar meningiomas account for approximately 5%-10% of all intracranial meningiomas and pose a distinct surgical challenge due to their deep-seated location and proximity to vital neurovascular and endocrine structures [1]. Based on preoperative imaging, particularly MRI, these tumors can be classified into three anatomically and surgically relevant subtypes: planum sphenoidale meningiomas, tuberculum sellae meningiomas (TSM)—the most frequently encountered variant—and the rarer diaphragma sellae meningiomas [2].

Visual disturbances are the most common presenting symptoms in patients with TSM, primarily due to compression of the optic apparatus. However, in less frequent cases, TSM may also impact pituitary function by compressing the pituitary gland, infundibulum, or hypothalamus, resulting in a range of endocrine dysfunctions [3]. Although clinically overt adrenal insufficiency is uncommon, subclinical impairments of adrenocorticotropin (ACTH) secretion from the anterior pituitary gland have been observed, especially in patients with larger tumors [4].

MRI remains the diagnostic gold standard, offering high-resolution visualization of the tumor’s size, growth pattern, and its relationship to adjacent structures. Dynamic hormonal testing, including pituitary provocation tests, is often necessary to assess both preoperative and postoperative pituitary function [5]. While surgical resection is the mainstay of treatment, thorough endocrinological evaluation remains essential to detect and manage any associated pituitary dysfunction. We report such a case of TSM that initially posed differential diagnostic challenges resembling a stroke.

## Case presentation

A 71-year-old male patient was admitted to our hospital for surgical management of a symptomatic TSM. He had a history of arterial hypertension, which had been well-controlled with antihypertensive medication. However, he recently developed persistent arterial hypotension following two intra-articular triamcinolone injections intended to treat chronic knee and shoulder pain. Subsequently, the patient experienced increasing fatigue, malaise, generalized weakness, and excessive tiredness. The onset of transient ischemic attack-like symptoms, including slurred speech and pronation drift in the left arm, led to admission to an external hospital to exclude a cerebrovascular event. Of note, he had preexisting visual impairments in his right eye due to a retinal gliosis.

**Fig. 1:**
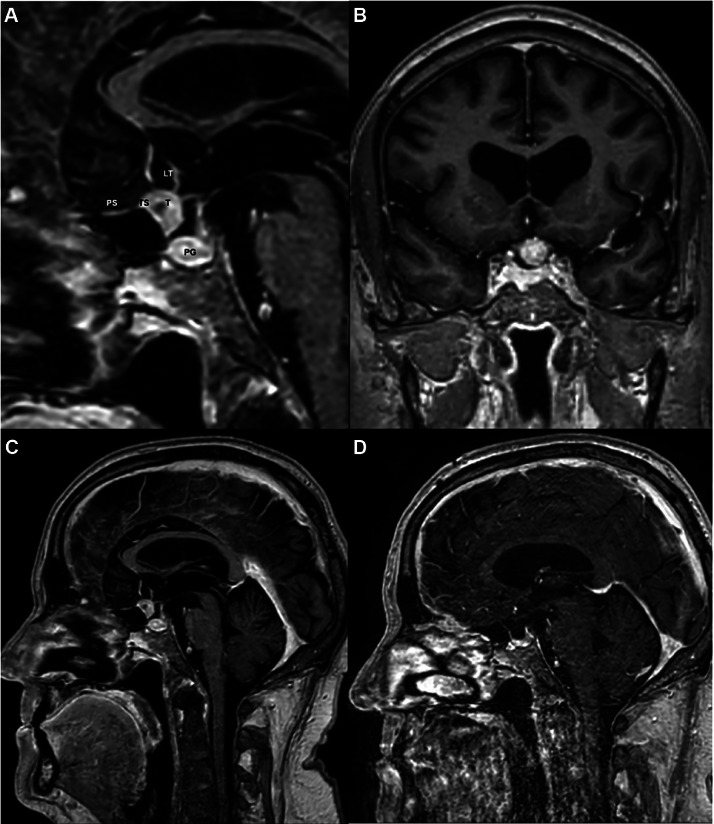
Preoperative and postoperative MRI scans of a symptomatic tuberculum sellae meningioma. (A) Sagittal magnified and (B) coronal contrast-enhanced T1-weighted MRI images demonstrating a well-circumscribed, contrast-enhancing lesion measuring approximately 1.2 cm at the tuberculum sellae bilaterally adjacent to the optic nerves, optic recess, lamina terminalis and anterior pituitary gland. (C) Preoperative and (D) postoperative sagittal contrast-enhanced T1-weighted MRI showing complete resection of the tuberculum sellae meningioma. LT, Lamina terminalis; PG, Pituitary gland; PS, Planum sphenoidale; T, Tumor; TS, Tuberculum sellae.

Cranial CT and CT angiography did not reveal any signs of a stroke or vascular occlusion, but did show potentially hemodynamically relevant moderate to severe stenoses of the right internal carotid artery, both extracranially near the bifurcation and intracranially above the ophthalmic segment. Additional significant findings included a partially calcified tumor at the tuberculum sellae. Cranial MRI revealed the TSM with bilateral compression of the optic nerves, close proximity to the anterior pituitary gland, and extension along the lamina terminalis, the anterior boundary of the hypothalamus (Fig. 1A-C). Endocrinological evaluation demonstrated reduced serum cortisol levels, an inadequate cortisol response to stimulation testing, and suppressed ACTH levels, consistent with secondary adrenal insufficiency. Due to the retinal gliosis, the cause of visual impairment in the right eye could not be conclusively attributed to tumor-related compression. Hydrocortisone replacement therapy was promptly initiated, leading to marked clinical improvement. Subsequently, the patient underwent resection of the TSM in the neurosurgical department of our hospital. Postoperative cranial MRI confirmed that the TSM was completely removed without damage to the surrounding structures (Fig. 1D).

## Discussion

We present a rare case in which a relatively small TSM contributed to hypothalamic-pituitary-adrenal (HPA) axis dysfunction, with secondary adrenal insufficiency likely exacerbated by recent intra-articular triamcinolone injections.

Systematic endocrine assessments are infrequently performed in the preoperative and postoperative management of suprasellar meningiomas [6]. Reported rates of preoperative hormonal abnormalities in these tumors vary widely, ranging from 0% to 42% [3,6,7]. This variability may largely be attributed to inconsistencies in the diagnostic methods and criteria used to evaluate anterior pituitary function.

TSMs are rarely associated with pituitary dysfunction, typically from mass effect on the pituitary gland, infundibulum, or hypothalamus - and usually in the context of larger lesions [4]. In a study by Bassiouni et al. [3], patients with endocrine disturbances had a mean tumor diameter of 3.4 cm (ranging from 2 to 6 cm), suggesting that a substantial tumor volume is generally necessary for hormonal compromise. Notably, in our case, the meningioma measured only 1.2 cm, yet it still contributed to clinically relevant hormonal dysfunction. This suggests that even small TSM can impair endocrine regulation under certain anatomical or pathophysiological conditions, underscoring the need for comprehensive hormonal workup regardless of tumor size.

Corticosteroid injections, such as triamcinolone, are widely used in musculoskeletal medicine for their localized anti-inflammatory effects. However, systemic absorption, particularly from depot formulations, can potentially lead to HPA axis suppression [8]. Recent studies and case reports have shown that even single intra-articular corticosteroid injections may induce secondary adrenal insufficiency, particularly in vulnerable individuals [9,10]. In the present case, it is plausible that the TSM had already caused subclinical ACTH deficiency, rendered the patient more vulnerable to the systemic effects of corticosteroids. The combined impact of pituitary dysfunction and exogenous steroid-induced suppression likely culminated in overt adrenal insufficiency, manifesting as fatigue, generalized malaise, and arterial hypotension. The latter condition, along with the significant stenoses of the right internal carotid artery, likely contributed to the symptoms of a transient ischemic attack.

## Conclusion

In conclusion, this case highlights that even small TSMs can disrupt pituitary function and that the risk may be amplified by systemic corticosteroid exposure. A high index of suspicion is essential, particularly when administering corticosteroids to patients with known or suspected sellar or parasellar lesions. Routine endocrine evaluation, vigilant monitoring, and close interdisciplinary collaboration among neurosurgery, neuroradiology, endocrinology, and ophthalmology are essential to prevent delayed diagnosis and ensuring optimal management.

## Declaration of generative AI and AI-assisted technologies in the writing process

During the preparation of this work the author(s) used Chat GPT in order to improve readability and language. After using this tool/service, the author(s) reviewed and edited the content as needed and take(s) full responsibility for the content of the publication.

## Patient consent

I confirm that a written, informed consent for publication of their case was obtained from the patient.
